# α-Glucosidase Inhibition and Antibacterial Activity of Secondary Metabolites from the Ecuadorian Species *Clinopodium taxifolium* (Kunth) Govaerts

**DOI:** 10.3390/molecules23010146

**Published:** 2018-01-11

**Authors:** Vladimir Morocho, Andrea Valle, Jessica García, Gianluca Gilardoni, Luis Cartuche, Alírica I. Suárez

**Affiliations:** 1 Departamento de Química y Ciencias Exactas, Universidad Técnica Particular de Loja (UTPL), Loja 1101608, Ecuador; andy171992@hotmail.es (A.V.); jkgarcia3@utpl.edu.ec (J.G.); gianluca.gilardoni@gmail.com (G.G.); lecartuche@utpl.edu.ec (L.C.); alirica1@yahoo.es (A.I.S.); 2 Facultad de Farmacia, Universidad Central de Venezuela, Caracas 1040, Venezuela

**Keywords:** *Clinopodium taxifolium*, ursolic acid, essential oil, Ecuador, *Klebsiella pneumoniae*, α-glucosidase

## Abstract

The phytochemical investigation of both volatile and fixed metabolites of *Clinopodium taxifolium* (Kunth) Govaerts (Lamiaceae) was performed for the first time. It allowed the isolation and characterization of the essential oil and six known compounds: carvacrol (**1**), squalane (**2**), uvaol (**3**), erythrodiol (**4**), ursolic acid (**5**), and salvigenin (**6**). Their structures were identified and characterized by Nuclear Magnetic Resonance (NMR) and Gas Chromatography coupled to Mass Spectroscopy (GC-MS), and corroborated by literature. The essential oil of the leaves was obtained by hydrodistillation in two different periods and analyzed by GC-MS and GC coupled to Flame Ionization Detector (GC-FID). A total of 54 compounds were detected, of which 42 were identified (including trace constituents). The major constituents were carvacrol methyl ether (18.9–23.2%), carvacrol (13.8–16.3%) and, carvacryl acetate (11.4–4.8%). The antibacterial activities were determined as Minimum Inhibition Concentration (MIC) against *Staphylococcus aureus*, *Enterococcus faecalis*, *Escherichia coli*, *Klebsiella pneumoniae*, *Proteus vulgaris*, *Pseudomonas aeruginosa* and *Micrococcus luteus*. The hexane and methanol extracts exhibited activity only against *Klebsiella pneumoniae* (250 and 500 μg/mL respectively), while the ethyl acetate extract was inactive. The hypoglycemic activity was evaluated by the in vitro inhibition of α-glucosidase. The ethyl acetate (EtOAc) extract showed strong inhibitory activity with IC_50_ = 24.88 µg/mL, however methanolic and hexanic extracts showed weak activity. As a pure compound, only ursolic acid showed a strong inhibitory activity, with IC_50_ = 72.71 μM.

## 1. Introduction

Since ancient times plants have generated great interest in every civilization, due to their potential as drugs. Today, they are still a very rich source of inspiration for the discovery of new active principles or for the study of biological activities [1,2].

Many methods are known to identify bioactive molecules from plant extracts. A very common approach is the so-called bioguided fractionation. It consists of setting up a fractionation scheme, and to screen fractions for the presence of the desired bioactive properties. Active fractions are further sub-fractionated and tested, until pure molecules responsible for the bioactivity can be identified. By the common procedures of thin-layer chromatography and column chromatography, coupled to spectroscopic techniques, the metabolites (contained in fractions previously identified as biologically active) can be characterized. The present study was performed by applying this procedure to anti α-glucosidase compounds in the Ecuadorian plant *Clinopodium taxifolium* (Kunth) Govaerts.

The choice of α-glucosidase as a molecular target of active compounds has been determined by the diffusion of diabetes as a common disease in Ecuador. According to official data of the INEC (National Institute of Statistics and Census of Ecuador), referred to 2016 [3], diabetes is the second cause of death in the country. The incidence of diabetes as cause of mortality is rapidly increasing, as it grew by 51% during the last ten years. Hence, the discovery of new active principles for anti-diabetes drugs can be considered a health priority. In particular, the presence of active compounds in natural sources is of great interest, because the use of natural products is very common in Ecuador. In fact, due to the high price of imported drugs, medicinal plants are an important complement to allopathic medicine, especially in countryside.

Besides anti-diabetes molecules, others secondary metabolites have been investigated, in order to determine the presence of new chemical structures or different biological activities. In particular, new volatile fractions are always an interesting component of plants, due to healthy applications and occasionally commercial interest of these products. For the reasons previously explained, a biological active essential oil is a good target for natural medicine. Many essential oils have been described for a wide range of biological activities [4]. In particular, anti-bacterial and anti-fungal properties are the most common, making the essential oils potential constituents for topic antiseptic products.

Several plants of the genus *Clinopodium* have been chemically investigated: flavonoids [5], other phenolics [6], caffeic acid oligomers [7,8], saponines and triterpenes [9] are common in these species. Metabolites like pterocarpanes [10] and lignans [9] are also known in some individuals.

*Clinopodium taxifolium* (Kunth) Govaerts is a native shrub of the Andes that grows between 1500 and 3000 m above sea level, in the provinces of Azuay, Loja and El Oro (Ecuador). It is known as Culantrillo de Cerro or Polea de Castilla. It is used as infusion in folk medicine to treat internal inflammations, flatulence, stomach pain, malaria and cough [11].

## 2. Results and Discussion

### 2.1. Characterization of Compounds ***1**–**6***

In this investigation, a bio-guided fractionation of the ethyl acetate (EtOAc) extract of *C. taxifolium* led to the isolation of ursolic acid (**5**), as the compound responsible for the α-glucosidase inhibition activity. This is an ubiquitous triterpene, well known for its wide spectra of biological activities including anticancer, anti-inflammatory [12], antimicrobial [13], anti-HIV [14], analgesic [15], diuretic [16], and others [17]. Recently, antidiabetic activity of acetyl ursolic acid has been reported [18], and the acid triterpene (**5**) had been recognized as an α-glucosidase inhibitor in a series of medicinal plants [19] as well a series of derivatives of this acid [20].

**Figure 1:**
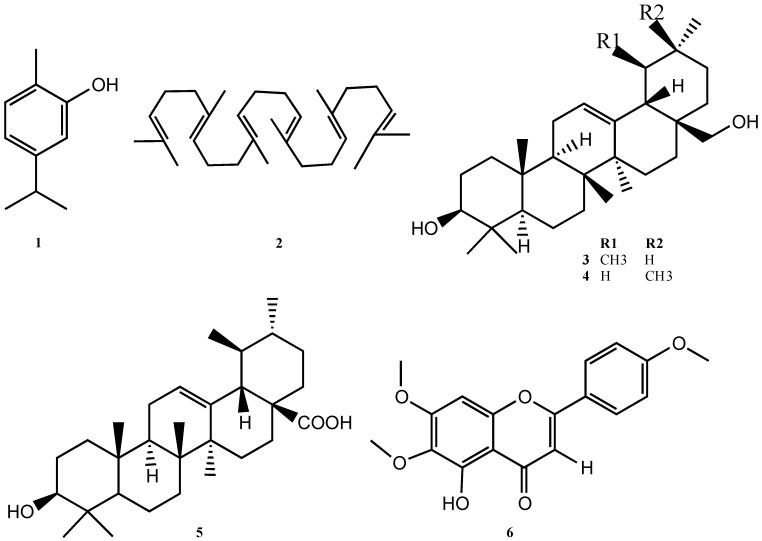
Structure of compounds (**1**–**6**) isolated from *Clinopodium taxifolium*.

The compounds were purified by chromatographic techniques, affording six metabolites (Figure 1). All molecules were characterized by spectroscopic techniques such as MS, NMR (^1^H and ^13^C) in one and 2D dimension experiments, and further comparison with literature data. Carvacrol was also identified by co-injection in GC/MS with an original standard.

Carvacrol (**1**). Yellowish oil; C_10_H_14_O; EI-MS *m*/*z* 150 [M]+ (38), 135 (100), 117 (5), 107 (9), 91 (14), 77 (6), 65 (2), 51 (2).

Squalene (**2**). Colorless oil; C_30_H_50_; ^1^H NMR (400 MHz, CDCl_3_) δ 1.55–1.68 (24 H, brs), 1.97–2.07 (20H, m), 5.07–5.14 (6H, m); ^13^C NMR (100 MHz, CDCl3) δ 136.0 (C-1), 135.0 (C-2), 131.4 (C-3), 124.5 (C-4), 124.4 (C-5), 124.3 (C-6), 39.9 (C-7), 39.8 (C-8), 28.4 (C-9), 26.9 (C-10), 26.8 (C-11), 25.8 (C-12), 17.8 (C-13), 16.2 (C-14), 16.1 (C-15). 

Uvaol (**3**). White amorphous powder; C_30_H_50_O_2_; ^1^H NMR (400 MHz, CDCl_3_) δ 5.12 (1H, t, *J* = 3.6 Hz, H-12), 3.51 (1H, d, *J* = 10.8 Hz, H-28b), 3.21 (1H, d, *J* = 10.8 Hz, H-28a), 3.17 (1H, dd, *J* = 11.0, 5.1 Hz, H-3), 1.09 (3H, s, H-27), 0.99 (3H, s, H-26), 0.94 (3H, s, H-23), 0.93 (3H, d, *J* = 5.2 Hz, H-30), 0.80 (3H, d, *J* = 5.6 Hz, H-29), 0.79 (3H, s, H-24), 0.78 (3H, s, H-25); ^13^C NMR (100 MHz, CDCl_3_) δ 39.5 (C-1), 27.4 (C-2), 79.1 (C-3), 38.9 (C-4), 54.2 (C-5), 18.4 (C-6), 32.9 (C-7), 40.1 (C-8), 47.8 (C-9), 37.0 (C-10), 23.5 (C-11), 125.2 (C-12), 138.5 (C-13), 42.2 (C-14), 26.2 (C-15), 23.3 (C-16), 35.3 (C-17), 55.3 (C-18), 39.6 (C-19), 39.5 (C-20), 30.7 (C-21), 39.5 (C-22), 28.2 (C-23), 16.9 (C-24), 16.7 (C-25), 15.6 (C-26), 23.4 (C-27), 70.1 (C-28), 17.5 (C-29), 21.5 (C-30).

Erythrodiol (**4**). White amorphous powder; C_30_H_50_O_2_; ^1^H NMR (400 MHz, CDCl_3_) δ 5.24 (1H, t, *J* = 3.0 Hz, H-12), 3.54 (1H, d, *J* = 10.6 Hz, H-28b), 3.21 (1H, d, *J* = 10.6 Hz, H-28a), 3.17 (1H, dd, *J* = 11.1, 5.1 Hz, H-3), 1.21 (3H, s, Me-30), 0.98 (3H, s, Me-27), 0.93 (3H, s, Me-29), 0.90 (3H, s, Me-23), 0.88 (3H, s, Me-26), 0.86 (3H, s, H-25), 0.77 (3H, s, Me-24). ^13^C NMR (100 MHz, CDCl3) δ 38.7 (C-1), 27.3 (C-2), 79.2 (C-3), 38.8 (C-4), 54.2 (C-5), 18.5 (C-6), 32.7 (C-7), 39.6 (C-8), 47.7 (C-9), 37.0 (C-10), 23.4 (C-11), 122.3 (C-12), 144.2 (C-13), 47.7 (C-14), 26.1 (C-15), 23.5 (C-16), 37.0 (C-17), 42.2 (C-18), 46.6 (C-19), 31.0 (C-20), 35.3 (C-21), 30.7 (C-22), 28.2 (C-23), 15.7 (C-24), 15.8 (C-25), 15.6 (C-26), 26.1 (C-27), 69.9 (C-28), 33.1 (C-29), 23.7 (C-30).

Ursolic acid (**5**). White powder; C_30_H_48_O_3_; ^1^H NMR (400 MHz, CDCl_3_) δ 5.24 (1H, t, *J* = 3.6, H-12), 3.21 (1H, dd, *J* = 10.0, 5.6 Hz, H-3), 2.17 (1H, d, *J* = 11.0 Hz, H-18), 1.25 (3H, s, H-26), 1.20 (3H, s, Me-23), 1.17 (3H, s, Me-27), 0.97(3H, s, Me-24), 0.96 (3H, d, *J* = 6.0 Hz, Me-29), 0.90 (3H, d, *J* = 6.0, Me-30), 0.78 (3H, s, Me-25); ^13^C NMR (100MHz, CDCl3): δ 39.2 (C-1), 29.8 (C-2), 79.2 (C-3), 38.7 (C-4), 55.4 (C-5), 18.4 (C-6), 33.1 (C-7), 39.6 (C-8), 48.1 (C-9), 37.2(C-10), 23.7 (C-11), 126.1 (C-12), 138.1 (C-13), 42.1 (C-14), 29.5 (C-15), 24.3 (C-16), 47.7 (C-17), 52.8 (C-18), 39.2 (C-19), 39.2 (C-20), 30.7 (C-21), 37.2 (C-22), 28.1 (C-23), 17.3 (C-24), 15.7 (C-25), 15.7 (C-26), 23.4 (C-27), 181.6 (C-28), 17.2 (C-29), 21.3 (C-30).

Salvigenin (**6**). Yellow powder; C_18_H_16_O_6_; ^1^H NMR (400 MHz, CDCl_3_) δ 12.77 (1H, s, 5-OH), 7.85 (2H, d, *J* = 9.2, H-2′, H-6′), 7.02 (2H, d, *J* = 9.2, H-3′, H-5′), 6.59 (1H, s, H-3), 6.55 (1H, s, H-8), 3.97 (3H, s, 7-OMe), 3.92 (3H, s, 6-OMe), 3.87 (3H, s, 4′-OMe); ^13^C NMR (100 MHz, CDCl3): δ 182.8 (C-4), 164.2 (C-2), 162.8 (C-4′), 158.9 (C-7), 153.2 (C-9), 153.4 (C-5), 131.9 (C-6), 128.0 (C-2′,C-6′), 123.7 (C-1′), 114.7 (C-3′,C-5′), 106.3 (C-5), 104.3 (C-3), 90.7 (C-10), 60.9 (6-OMe), 55.7 (7-OMe), 55.6 (4′-OMe).

### 2.2. Essential Oil Analysis

The distilled volatile fraction was obtained with a yield of 0.33 ± 0.11% (*w*/*w*) for the collection of 2015 and 0.73 ± 0.11% (*w*/*w*) for the one of 2016. The mean relative density of the two series was *d*_20_ = 0.953 ± 0.001 g/cm^3^ and *d*_20_ = 0.953 ± 0.001 g/cm^3^ respectively, while the mean refractive index was *n*_20_ = 1.499 ± 0.002 and *n*_20_ = 1.502 ± 0.001 respectively. In the essential of *C. taxifolium*, 54 compounds were detected, of which 42 were identified. Oxygenated monoterpenes were major constituents, corresponding to more than 50% of the entire mixture. Oxygenated sesquiterpenes were also a significant fraction, while unknown compounds only represented about 2%.

Whereas the quantitative composition of almost all the constituents of the essential oil were quite constant in both collection years, the oxygenated sesquiterpenes were quite variable, varying about between 24% in 2015 and 8% in 2016. Despite the collection being performed both times during the same months, a climatic factor is not the most probable cause of the variation, as the samples were obtained from the same spots. This variability is quite interesting, as the amount of the oxygenated sesquiterpenic fraction is sometimes related to the value of an essential oil. Elemol and agarospirol are the most variable oxygenated sesquiterpenes, with the first one changing from about 9% in 2015 to less than 5% in 2016, and the second one from 8% in 2015 to traces in 2016. According to recent literature [21,22,23], the agarospirol amount in the essential oil of *Aquilaria malaccensis* is closely related to bacterial and fungal interactions in plant and soil. Despite the fact that this phenomenon cannot be demonstrated in the present work, it seems to be a possible ecologic hypothesis for future studies. The qualitative and quantitative chemical composition of the essential oil is presented in Table 1.

### 2.3. Hypoglycemic Effect and Antibacterial Activity

The hypoglycemic effect of the extracts and four of the isolated compounds (**4**–**7**, Table 2) were evaluated using α-glucosidase from *Saccharomyces cerevisiae* (G5003, Sigma, St. Luis., MO, USA). On one hand, the hexane and methanolic extracts exhibited a weak inhibitory effect while the ethyl acetate extract showed a strong inhibitory effect. On the other hand, only ursolic acid demonstrated to be the most promising compound with an IC_50_ below than 100 µM. Uvaol, salvigenin and squalene showed inhibitory effect at concentrations higher than 500 µM having a poor or no effect over the enzyme (Table 2). 

Ursolic acid (UA) is a natural terpene that has been isolated from a variety of plant species and has demonstrated a wide variety of interesting biological activities. Although it has no cytotoxic effect itself, UA has been considered as a chemopreventive agent that modulates cellular response for cancer prevention, having anti-inflamatory and anti-oxidative properties and also an ability to modulate processes occurring inside a tumor, leading to cell death [37]. 

According to [38], UA promotes glucose uptake in HepG2 cells and reduced the blood glucose levels of diabetic mice to a different extent even preventing the body weight loss caused by the induction with STZ. Besides, the a α-glucosidase inhibitory effect of UA was 510 µg/mL (1116.7 µM), fourteen times less effective compared to the value obtained in this study, which can be attributed to the short time of incubation (10 min), compared to our method that measures the inhibitory effect over a lapse of sixty minutes.

According to [39], MIC values ranging between 100 to 500 µg/mL have a moderate activity and MIC values higher than this are considered weak or inactive, so, only the hexanic extract exhibited moderate inhibition against *Klebsiella pneumoniae* (Table 3). Up to date, there is no research about the biological properties of *Clinopodium taxifolium*. Unfortunately, the antibacterial effect of the compounds was not tested in this work, due to the low yield obtained and also because the four compounds were mainly tested for hypoglycemic effect. However, according to literature, UA has a good profile of antibacterial activities showing good inhibition against *Klebsiella pneumoniae*, *Shigella flexneri*, *Escherichia coli* and *Staphylococcus aureus* with MIC values below than 100 µg/mL [40]. Besides, UA exerted synergistic effects by combining it with neomycin, amikacin, kanamycin and gentamicin, showing a broad spectrum of synergistic activity (MIC values 8 to 32 times higher than antibiotic alone) against *S. aureus*, *B. subtilis*, *E. coli*, *P aeruginosa*, *K. penumoniae*, *S. flexneri*, *L. monocytogenes* and *V. choleare*. Also, UA showed good inhibition activity against *M. tuberculosis* with a MIC value ranging from 10 to 20 µg/mL, measured by colorimetric resazurin assay [41].

## 3. Materials and Methods

### 3.1. General Information

The NMR spectra were run on a Varian (Walnut Creek, CA, USA, 400 MHz for ^1^H and 100 MHz for ^13^C) in CDCl_3_. Chemical shifts were reported in δ (ppm), relative to the signal of tetramethylsilane (TMS) and coupling constants (*J*) in Hz. The GC-MS analyses were performed on an Agilent Technologies (Wilmington, DE, USA) 6890N gas chromatograph coupled to a mass spectrometer detector Agilent Technologies 5973N. For both GC-MS and GC-FID analyses, the instrument was equipped with a DB5-MS Agilent 122-5532 column (length 30 m, internal diameter 0.25 mm thickness of the stationary phase 0.25 µm). Silica gel 60 (Merck KGaA, Darmstadt, Germany, from 0.063 to 0.200 mm) and RP-18 (Merck, KGaA, Darmstadt, Germany, 40–63 µm) were used as stationary phases for column chromatography. Normal phase Thin Layer Chromatography (TLC), with fluorescence indicator at 254 nm, were purchased by Sigma-Aldrich. After exposure to UV light (254 and 366 nm), the plates were revealed with a mixture of sulphuric acid and vanillin. All organic solvents were bought in Brenntag (Brengtan, Guayaquil, Ecuador) and re-distilled before using. Optical rotations were acquired in an Automatic Polarimeter (Jinan Hanon Instruments Co. Ltd., Jinan, China) MRC P810. Refractive indices were measured with a digital ABBE refractometer (Boeco, Hamburg, Germany).

### 3.2. Plant Material

The aerial parts of *Clinopodium taxifolium* in flowering state were collected at two times, February 2015 and February 2016, in the sector Cañicapac, Saraguro, Loja province, with coordinates 690820N and 9606032E, at 2586 m above sea level. The botanical sample was deposited in the herbarium of the UTPL with voucher number PPN-la-101.

### 3.3. Extraction and Isolation of Metabolites

Dry leaves of *C. taxifolium* (500 g) were ground to powder. The ground material was exhaustively submitted to solvent extraction by maceration at room temperature, by increasing solvent polarity during a 3 days process. Hexane (Hex), ethyl acetate (EtOAc), and methanol (MeOH) were used in this order. After solvent removal at reduced pressure, three dry extracts were obtained: hexanic extract (3.61 g) with a yield of 0.62% on dry material, ethyl acetate (25.79 g) with a yield of 5.16% and methanol (34.63 g) with a yield of 6.93%. Column chromatography was performed on 3 g of hexane extract. Silica gel (300 g) was packed in a column (50 mm × 800 mm) as stationary phase and it was eluted with a mixture of n-hexane/EtOAc (325 fractions of 25 mL each), according to an increasing gradient of eluotropic strength (from 10:90 to 0:100). The fractions were pooled according to TLC profiles (normal phase TLC, eluted with n-hexane/EtOAc according to metabolite polarity). A fraction, denominated AR002/5 (597.7 mg), was submitted to further fractionations on normal phase, with different isocratic mixtures of n-hexane/EtOAc and CH_2_Cl_2_/cyclohexane, affording two pure compounds. One of them was identified by GC/MS as carvacrol (**1**) (0.7 mg) [42], the other one as squalene (**2**) (6 mg) [43]. 

The EtOAc extract (2 g) was submitted to normal phase CC (150 g of silica). The stationary phase was packed in a column (50 mm × 400 mm) that was eluted with a mixture of n-hexane/EtOAc (230 fractions of 15 mL each), according to increasing polarity gradient (from 90:10 to 0:100). Finally, the column was washed down with pure MeOH. The fractions were combined according to their TLC patterns (normal phase TLC, eluted with n-hexane/EtOAc according to metabolite polarity). A subsequent gradient fractionation of an 18 mg fraction, performed with n-hexane/EtOAc, afforded two metabolites. The first one (8 mg) was a mixture of two triterpenes: uvaol (**3**) and erythrodiol (**4**) [44], the second one (7 mg) was identified as pure uvaol (**3**) [45].

The most polar fraction of the same extract (18 mg) was successively chromatographed on silica gel (2 g in a column of 10 mm × 100 mm), eluted with a mixture of n-hexane/EtOAc (40 fractions of 1 mL each). The elution was performed according to increasing polarity with a mixture of n-hexane:EtOAc (from 90:10 to 70:30), obtaining two main fractions. One of these (5 mg) afforded the triterpene ursolic acid (**5**) [46].

The methanol extract (5 g) was submitted to a process for removing chlorophyll by partition between MeOH/H_2_O (90:10) and hexane, repeated for three times. After evaporation of the polar phase, 1 g of the residue was fractionated by CC over silica gel (100 g in a column of 30 mm × 400 mm), eluting according to a polarity gradient of EtOAc/MeOH/H_2_O (from 150:15:5 to 50:15:5). The process afforded 100 fractions of 10 mL each. The fractions were pooled according to TLC profiles (normal phase TLC, eluted with n-hexane/EtOAc according to metabolite polarity). Further purification of a fraction (108 mg) on CC (11 g of silica gel in a column of 20 mm × 200 mm), with n-hexane/EtOAc (from 80:20 to 40:60), yielded the flavonoid salvigenin (**6**) (10 mg).

### 3.4. Distillation and Analysis of the Essential Oil

The volatile fraction was obtained by hydrodistillation of 250 g of dry aerial parts. The dry plant material was previously re-hydrated in water for 1 h and distilled in the same water during 4 h. The distillation was performed in four repetitions for each collection and the analytical results expressed as mean values and standard deviation.

The qualitative analysis was achieved by GC-MS and the constituents of the essential oil determined by comparison of mass spectra and linear retention indices (LRI) with literature [24]. Linear Retention Indices (LRI) were calculated according to Van Den Dool and Kratz [47].

The quantitative analysis was performed by GC-FID, without applying any response factor but normalizing data with internal standard (nonane). For both GC-MS and GC-FID analyses, the analytical conditions were as follow: the sample was diluted at 10% (*v*/*v*) in cyclohexane, the injection volume was 1:1 with auto-injection system, helium (flow 1 mL/min) was the carrier gas, the injector was operated in split mode (ratio 10:1) at a temperature of 250 °C, the oven was programmed with an initial temperature of 50 °C for 1 min, then increased to 270 °C at 10 °C/min and kept at 270 °C for 25 min.

### 3.5. Minimum Inhibitory Concentration (MIC) Determination

MIC values were determined by the microdilution broth method using a final concentration of 5 × 105 CFU/mL. Seven strains of bacteria from ATCC (Medibac, Quito, Ecuador), three gram positive and four gram-negative bacteria were used for the assay. MIC was defined as the lowest concentration of substance that prevents visible growth of the organism in the microdilution wells (CLSI, M7-A7 2006). DMSO solutions of the samples were prepared at a concentration of 20 μg/mL for extracts and 200 μL/mL for essential oils. The assays were carried-out in 96-well plates (Eppendorf AG, Hamburg, Germany) and two-fold serial dilution was employed, to obtain decreasing concentrations of 1000–7.81 μg/mL (extracts) and 10,000–70.81 μg/mL. Incubation was at 37 °C for 24 h [48]. Gentamicin was used a positive control with a MIC value of 0.40 μg/mL except for *E. faecalis* where tetracycline was used (MIC 1.95 μg/mL).

### 3.6. α-Glucosidase Inhibition Assay

α-Glucosidase inhibitory activity was determined using a 96-well microtiter plate, with *p*-nitrophenyl-α-d-glucopyranoside (PNPG, SIGMA N1377) as the substrate, according to a method describe in literature [49], with slight modifications. Sample solutions were prepared by dissolving 10 mg in 1 mL MeOH:H_2_O (1:1). Dilutions in Phosphate Buffered Saline (PBS) were made in case of getting complete enzyme inhibition. First, 75 μL of PBS (SIGMA-P4417) were mixed with 5 μL of the sample and 20 μL of the enzyme solution (SIGMA G5003, 0.15 U/mL in PBS pH 7.4). Then, it was pre-incubated at 37 °C for 5 min prior to the initiation of the reaction by adding the substrate. After pre-incubation, 20 μL of PNPG (5 mM in phosphate buffer, pH 7.4) was added and then incubated at 37 °C. The amount of p-NP released was measured in an EPOCH 2 (BIOTEK^®^, Biotek Instruments Inc., Winooski, VT, USA) microplate reader at 405 nm for 60 min, recording the absorbance every 5 min. The results were expressed as inhibition percentage by means of the formula described by [50] as follows:

Inhibition(%)=[(Ao−As)/Ao]×100

where Ao is the absorbance recorded for the enzymatic activity without inhibitor (control), and As is the absorbance recorded for the enzymatic activity in presence of the inhibitor (sample). IC_50_ value was calculated by curve fitting of data (GraphPad Prism 5.0, GraphPad Software, Inc., La Jolla, CA, USA). Acarbose was used as positive control.

## 4. Conclusions

Finally, and after discuss the showed results, it is important to point out few conclusions in this work. First, the fact that this investigation was realized in view that some organizations like the World Health Organization (WHO) encourage the use of herbal medicines for some diseases like diabetes, especially in a country like Ecuador with wide use of medicinal plants. The phytochemical investigation of organic extracts was successfully carried out. Six known compounds were isolated and characterized from the aerial parts of the species. The identification of triterpenes, among others, as the major compounds present in *C. taxifolium* is consistent with reports from other species of this genus. Unfortunately, none of the isolated metabolites showed antibacterial activity. However, the results with α-glucosidase inhibition clearly encourage further studies with this plant. The fact that the EtOAc fraction, from which was isolated the ursolic acid (5), showed the best IC_50_, inclusive compared with the Acarbose, indicate a possible synergic effect of the different compounds present in this fraction. Further studies will be necessary to characterize minor components, which could be also responsible of the strong α-glucosidase inhibition showed.

## Figures and Tables

**Table 1:** Chemical composition of the essential oil distilled from *C. taxifolium*.

Compound	Calculated LRI *	Literature LRI *	2015		2016		Mean Values on Two Years		Reference Literature for LRI
			% FID	σ	% FID	σ	% FID	σ	
α-Pinene	947	939	0.77	0.33	0.90	0.35	0.84	0.31	[24]
Sabinene	981	975	0.71	0.27	0.32	0.28	0.51	0.33	[24]
Alpha terpinene	1014	1017	0.16	0.04	0.18	0.07	0.17	0.06	[24]
*p*-Cymene	1020	1024	2.57	0.85	4.85	111	3.71	1.53	[24]
γ-Terpinene	1049	1059	0.73	0.19	0.92	0.35	0.83	0.27	[24]
Trans-sabinene hydrate	1059	1070	0.27	0.09	0.38	0.03	0.32	0.08	[24]
Linalool	1091	1096	0.15	0.05	Trace	-	0.08	0.09	[24]
Pinocarvone	1148	1160	0.10	0.01	Trace	-	0.05	0.06	[25]
3-Pinanone	1164	1168	3.82	1.11	1.84	1.73	2.83	1.69	[26]
4-Carvomenthenol	1170	1177	0.24	0.11	0.23	0.06	0.23	0.08	[27]
Dihydro carveol	1186	1194	0.43	0.14	0.29	0.10	0.36	0.13	[24]
Thymol methyl ether	1227	1235	0.93	0.29	1.30	0.40	1.11	0.37	[28]
Carvacrol methyl ether	1248	1244	18.87	3.89	23.35	5.66	21,11	4.99	[24]
Methyl carvacrol	1254	1248	Trace	-	0.13	0.05	0.06	0.08	[29]
Piperitone	1248	1252	0.25	0.10	Trace	-	0.13	0.15	[24]
Methylbenzoate	1268	1271	0.12	0.03	Trace	-	0.06	0.07	[30]
Endobornyl acetate	1281	1285	0.21	0.04	Trace	-	0.10	0.12	[24]
trans-pinocarvyl acetato	1294	1298	2.13	0.46	0.66	0.91	1.39	1.03	[24]
Carvacrol	1308	1299	13.81	2.01	16.26	0.65	15.04	1.89	[24]
Myrtenyl acetate	1325	1326	0.91	0.16	0.42	0.19	0.66	0.31	[24]
Piperitenone	1334	1343	0.16	0.02	Trace	-	0.08	0.09	[24]
δ-Elemene	1337	1338	1.10	0.17	Trace	-	0.55	0.61	[24]
Unidentified	1353	-	Trace	-	1.40	0.29	0.70	0.79	-
Carvacryl acetate	1373	1371	11.35	1.42	14.82	1.66	13.08	2.35	[31]
β-Bourbonene	1379	1388	0.22	0.06	0.27	0.05	0.25	0.05	[24]
Unidentified	1387	-	Trace	-	0.07	0.06	0.04	0.06	-
β-Elemene	1389	1390	0.21	0.05	0.12	0.01	0.16	0.06	[24]
Z-caryophyllene	1414	1419	3.21	0.54	3.05	0.27	3.13	0.40	[24]
Unidentified	1428	-	0.09	0.03	0.11	0.02	0.10	0.02	-
α-humulene	1448	1449	Trace	-	0.12	0.01	0.06	0.06	[32]
Aromadendrene	1452	1447	0.11	0.01	0.12	0.01	0.12	0.01	[33]
Unidentified	1455	-	0.09	0.02	Trace	-	0.04	0.05	-
*trans*-β-farnesene	1458	1456	0.09	0.02	Trace	-	0.04	0.05	[24]
Unidentified	1468	-	Trace	-	Trace	-	Trace	-	-
Germacrene D	1474	1484	1.59	0.27	1.81	0.16	1.70	0.23	[24]
β-selinene	1480	1486	Trace	-	Trace	-	Trace	-	[34]
Ledene	1484	1496	Trace	-	Trace	-	Trace	-	[24]
Unidentified	1487	-	0.73	0.08	0.79	0.07	0.76	0.08	-
Unidentified	1492	-	Trace	-	0.44	0.09	0.22	0.25	[24]
γ-cadinene	1505	1513	Trace	-	Trace	-	Trace	-	[27]
Unidentified	1508	-	Trace	-	Trace	-	Trace	-	-
δ-Cadinene	1513	1523	0.61	0.14	0.65	0.01	0.63	0.09	[24]
Cadina-1,4-diene	1524	1534	Trace	-	Trace	-	Trace	-	[24]
Selina-3,7(11)-dien	1530	1537	Trace	-	Trace	-	Trace	-	[35]
Elemol	1546	1549	9.05	2.60	4.79	1.24	6.92	2.96	[24]
Unidentified	1566	-	0.35	0.10	0.23	0.09	0.29	0.11	-
Unidentified	1577	-	0.79	0.28	0.55	0.22	0.67	0.26	-
Unidentified	1592	-	2.34	1.12	Trace	-	1.17	1.46	[24]
Guaiol	1601	1600	1.36	1.25	0.40	0.14	0.88	0.96	[24]
Agarospirol	1620	1631	8.02	3.89	Trace	-	4.01	5.03	[36]
β-Panasinsene	1626	1623	Trace	-	Trace	-	Trace	-	[24]
Alpha eudesmol	1635	1632	5.34	0.87	2.31	0.74	3.83	1.81	[24]
Hinesol	1644	1641	Trace	-	0.53	0.07	0.26	0.29	[24]
Selina-3,11-dien-6-a-ol	1650	1644	Trace	-	5.30	0.81	2.65	2.95	[24]
Hydrocarbon monoterpenes			4.93		7.18		6.06		
Oxygenated monoterpenes			53.62		59.67		56.64		
Hydrocarbon sesquiterpenes			7.14		6.13		6.64		
Oxygenated sesquiterpenes			23.77		7.50		15.64		
Unidentified			2.05		1.68		1.87		
Others			2.46		7.74		5.10		
**TOTAL**			**93.98**		**89.91**		**91.94**		

* LRI: Lineal Retention Index.

**Table 2:** In vitro α-glucosidase inhibitory effect (as IC_50_ values) of extracts and compounds isolated from *Clinopodium taxifolium*.

No.	Extract/Compound	α-Glucosidase
		IC_50_^†^
1	Hexane extract	228.7 ± 1.3
2	EtOAc extract	24.9 ± 0.8
3	MeOH extract	431.3 ± 2.5
4	Squalene	>1000
5	Ursolic acid	72.7 ± 0.9
6	Uvaol	521.0 ± 2.7
7	Salvigenin	>1000
8	Acarbose *	377.0 ± 1.9

^†^ IC_50_ values expressed as μg/mL for extracts and µM for pure compounds. * Acarbose was used as positive control.

**Table 3:** Minimum Inhibition Concentration (MIC) values of total extracts and essential oils from *C. taxifolium*.

Microorganims ATCC^®^	HEX. (mg/mL)	EtOAc (μg/mL)	MeOH (μg/mL)	Essential Oil 2015 (μg/mL)	Essential Oil 2016
*Staphylococcus aureus 25923* ^†^	-	-	-	5.00	5.00
*Enterococcus faecalis 19433* ^‡^	-	-	-	10.00	10.00
*Escherichia coli O157:H7* ^†^	-	-	-	1.95	-
*Klebsiella pneumoniae 9997* ^†^	0.25	-	0.50	1.95	-
*Proteus vulgaris 8427* ^†^	-	-	-	15.62	-
*Pseudomonas aeruginosa 27853* ^†^	-	1.00	-	15.62	-
*Micrococcus luteus 10240* ^†^	-	-	-	5.00	5.00

^†^ 1 mg/mL solution of Gentamicine was used as a positive control for all bacteria (except *E. faecalis*), exhibiting a MIC value less than 0.4 µg/mL. ^‡^ 5 mg/mL solution of tetracycline was used for *E. faecalis*, showing a MIC value of 1.95 µg/mL.
